# L-Shaped Coplanar Strip Dipole Antenna Sensor for Adulteration Detection

**DOI:** 10.3390/s25020506

**Published:** 2025-01-16

**Authors:** Sreedevi K. Menon, Massimo Donelli

**Affiliations:** 1 Department of Electronics and Communication Engineering, Amrita Vishwa Vidyapeetham, Amritapuri 690525, India; 2 Centre for Flexible Electronics and Advanced Materials, Amrita Vishwa Vidyapeetham, Amritapuri 690525, India; 3 Department of Civil Environmental and Mechanical Engineering, University of Trento, 38123 Trento, Italy; massimo.donelli@unitn.it

**Keywords:** adulteration sensor, coplanar strip dipole antenna, microwave sensor, sensitivity

## Abstract

The present study proposes an L-shaped coplanar strip dipole antenna for sensing the presence of adulterants in liquid food samples. The proposed antenna dimensions are optimized using ANSYS HFSS, and a prototype is fabricated and validated. The sensing region is optimized based on the current distribution and measured reflection coefficients. Adulterant detection is performed by monitoring the variation in the reflection coefficient and resonance frequency of the antenna sensor. To verify the effectiveness of the proposed planar dipole as a sensor, an adulterant, which is hydrogen peroxide, is added to various liquid samples – milk, pineapple juice, and mango juice. The reflection coefficient of the antenna sensor is found to vary with various concentrations of the samples in the study. The sensitivity analysis of the antenna sensor and the repeatability of the results is also analyzed in the work. The experimental analysis assures the use of the proposed antenna as a sensor for the detection of adulterants in liquid food samples.

## 1. Introduction

In the recent past, a wide range of microwave sensors have been utilized to address various new measurement challenges, making them increasingly prevalent in various sectors. The development of microwave sensors commenced in the 1950s, during which time there was a movement to improve the methods to measure permittivity and to investigate the relationship between permittivity and the physical properties of materials and compounds [1]. Considering this, a lot of applications of microwave sensors have been proposed in research over the years. Microwave radars for human detection [2], permittivity analysis of materials [3], corrosion detection [4,5], adulteration detection [6], glucose monitoring [7], strain sensing [8], structural health monitoring [9], breast cancer detection [10], and temperature sensing [11] are some of the areas in which these sensors are being used. In the above-mentioned sensors, printed resonant sensing devices are popular due to their small size and ease of integration. Configurations like patch antennas, metamaterials, resonators, and couplers have been widely used for microwave sensing. Some of the devices used in this sensing technology are discussed below.

By incorporating slots in the radiating patch and using symmetric short-circuit probes, an ultra-wideband circular polarized implantable patch antenna for blood glucose monitoring applications is designed [12]. The design also includes a superstrate layer that enhances biocompatibility and minimizes the antenna’s impact on the human body, which is essential for medical applications. A complementary curved ring resonator (CCRR) to measure the permittivity, or electrical properties, of different materials is proposed in [13]. When materials with different permittivity are placed near the sensor, it changes the frequency at which the sensor resonates, allowing for precise measurements, and the sensor has been shown to be highly sensitive and accurate in tests with various common materials. Recently, a planar microwave sensor designed using triangular complementary split ring resonators (CSRRs) for glucose detection was proposed [14]. A Cycle GAN-boosting algorithm as a learnable technique to improve the sensing performance of low-quality resonance-based sensor responses was also included in the work. A microstrip sensor with a 50 Ω microstrip line as the host region and a sensing area with an M-shaped element at 2.45 GHz has been reported [15]. An equivalent circuit model of the sensor was also deduced by the authors. Using inverse surrogate models and analytical correction techniques, CSRRs were optimized for tuning for industrial microwave sensors in [16]. The sensor effectively characterizes low-permittivity dielectric substrates, with a maximum relative error between simulated and measured results under 5.5%. A sensor designed using a microstrip line and a modified resonator to measure the electromagnetic properties of magnetodielectric (MD) materials is presented in [17]. The sensor determines both the permittivity and permeability of the materials through frequency compensation and artificial neural networks. An adulteration detection sensor using a microstrip closed loop antenna (MCLA) operating at 2.48 GHz is proposed by Aiswarya, S. et al. [18]. By testing different mixtures of milk with water and other chemicals, researchers can measure changes in frequency and signal strength to determine how much adulterant is present. A fluid-level sensor using a microstrip line and SMA oscillator to measure the height of liquids inside a closed metal pipe was proposed recently [19]. It works by sending radio frequency (RF) signals through a microstrip line, and as the fluid level changes, the variation in the signal strength is analyzed. 

A polyvinyl alcohol (PVA) coated complementary split ring resonator (CSRR) and stepped impedance resonator can be used for humidity detection [20]. The proposed sensor measures humidity levels between 25% and 85%, showing strong performance in both frequency and magnitude responses. An antenna sensor with defective ground structures operating in the dual band for wireless monitoring temperatures ranging from 25°C to 800°C is presented in [21]. A study on positioning the SRRs within the Fresnel zone of two printed Yagi-Uda antennas shows better sensitivity to changes in materials, making it effective for various applications [22]. A patch antenna combined with metamaterials to measure the dielectric properties of tiny samples without direct contact is discussed [23]. Research on sensor’s effectiveness and potential applications in studying biological materials and phenomena is presented by the authors. An inclinometer using a microstrip resonator, which changes its length as the angle changes, causing a shift in its resonance frequency, is demonstrated in [24]. The sensor has a sensitivity of 0.384 mm per degree and a resolution of 0.035 degrees, making it effective for measuring inclination angles.

Much recent research has focused on employing antennas and resonators as sensors to detect adulteration. A miniaturized antenna sensor resonating at 13.3 GHz is presented for the detection of adulteration in spices [25]. The adulterant detection is performed by monitoring the shift in the fundamental resonant frequency with dielectric permittivity. A multi-layer antenna sensor with an artificial magnetic conductor (AMC) array has recently been reported to detect the presence of water and ethanol in gasoline [26]. The authors also used the principle of resonant frequency shifts to detect adulteration. A multiple complementary split ring resonator for detecting water–ethanol mixture for the computation of the dielectric constant of liquids is reported [27]. For this purpose, the authors monitored the variation in S21(dB). A metamaterial-based resonator is employed in a non-destructive electromagnetic (EM) sensor system to identify and quantify chemical adulterants in cooking oils in the X-band [28]. A slot-loaded rectangular microstrip patch antenna (MPA) for detecting liquid chemicals with high relative permittivity and high loss tangent at 2.5 GHz is introduced in [29]. A cylindrical dielectric resonator antenna (CDRA), with HEM11 mode at 5.25 GHz for sensing isopropyl, ethanol, methanol, and water, is investigated in [30]. A circular substrate integrated waveguide (CSIW)-based biochemical detector for aqueous solvents was recently reported [31]. A negative-refractive-index transmission line metamaterial was used to design a planar microwave sensor operating at 2.5 GHz, which exhibits significant improvement in sensitivity and linearity [32].

This paper addresses the design and development of an L-dipole antenna sensor for adulteration detection. The antenna sensor suggested in this study can detect hydrogen peroxide (H_2_O_2_) in milk, mango juice, and pineapple juice. Numerical simulations followed by experiments are carried out by varying the concentration of liquid hydrogen peroxide in the above three samples. To determine the sensitivity, the resonant frequency shift is monitored for various adulterant quantities. The rest of this paper is organized as follows. Section 2 describes the detailed methodology of sensor design and experiments, whereas Section 3 analyses the antenna characteristics. Section 4 explains how the antenna operates as an adulteration sensor, followed by Section 5, Results and Discussions. Section 6 concludes this paper 

## 2. Methodology

**Figure 1:**
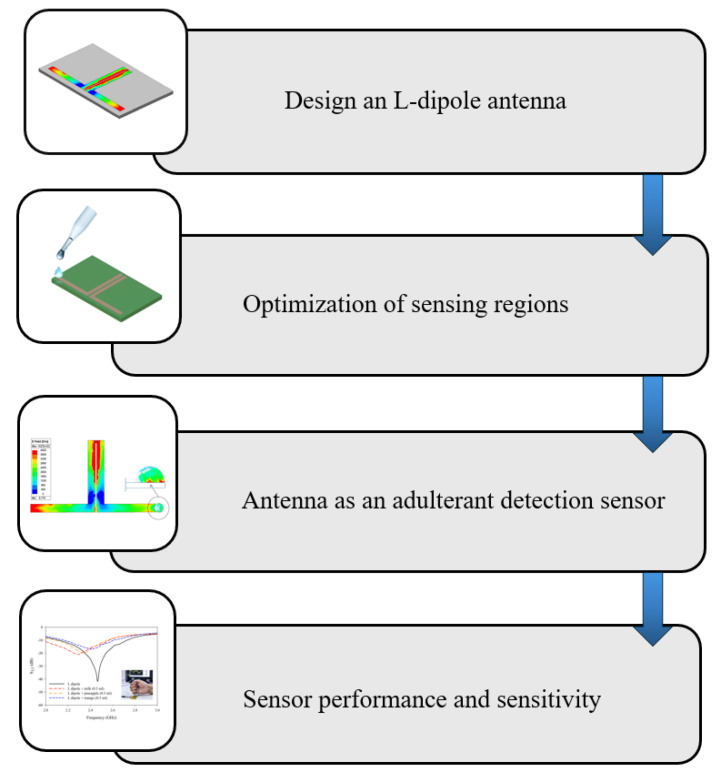
Methodology adopted in the present study.

This section provides the methodology adopted in the sensor design and the related measurements. The methodology adopted for the study is presented in Figure 1.

### 2.1. Antenna Design

The initial step is to design an L-shaped coplanar strip dipole antenna and characterize it for reflection and radiation properties. An FR4 epoxy substrate is selected, considering factors such as affordability, durability, and response to the stimuli. The next step is to optimize the sensor for the application. To test the tunability of the antenna, the dimensions were varied, and the effect on the resonant frequency was studied. For this, ANSYS HFSS simulation software (V 2023) was used, and the dimension of the antenna sensor to operate at 2.45 GHz was optimized. This frequency was selected to upgrade the sensor to a wireless module for wireless sensing. In the third stage, the sensor was fabricated using standard photolithography and etching techniques. In the next stage, the measurement of the fabricated antenna was carried out using Keysight N5227B PNA Network Analyser after standard calibration. The radiation characteristics were studied in the Anechoic chamber at Amritatrang at Amrita Vishwa Vidyapeetham. 

### 2.2. L Shaped Coplanar Strip Dipole Antenna as a Sensor

Once the characterization is completed, the effectiveness of the antenna as sensor is analyzed. *Test 1*: The sensing region is optimized by experiment using three different liquid samples (unadulterated samples of milk, pineapple juice and mango juice) repeated multiple times. The structure of the antenna is divided into 6 points; in each of the points, the response of the sensor for the samples is studied. *Test 2*: The different samples with and without adulterant (H_2_O_2_) are analyzed by proper placement in the sensing region. The required quantity of the sample is measured and placed using a micro-needle at the sensing region. *Test 3*: To ensure reliability and repeatability, each sample is analyzed multiple times. The antenna sensor characteristics are studied as a function of reflection with frequency. The results have been verified for reproducibility by fabricating multiple sensors and by conducting the experiments on separate days under the same conditions as well as same measurements on the sensor multiple times. This methodology enables us to comprehend the microwave sensor’s primary characteristics and its potential for use in a variety of liquid sensing applications. A comparative analysis of the sensors reported on the effectiveness of the proposed antenna as a liquid sensor.

## 3. Analysis of L-Shaped Coplanar Strip Dipole Antenna

**Figure 2:**
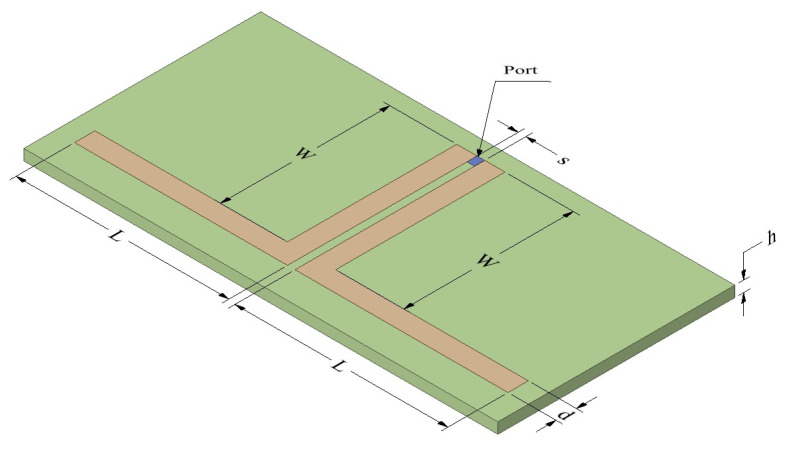
L-shaped coplanar strip dipole antenna.

L-shaped coplanar strip dipole antenna optimization and performance analysis are presented in this section. The dipole antenna is designed with two metallic conductors on a dielectric substrate, as illustrated in Figure 2. The two strips are electromagnetically coupled to each other.

**Figure 3:**
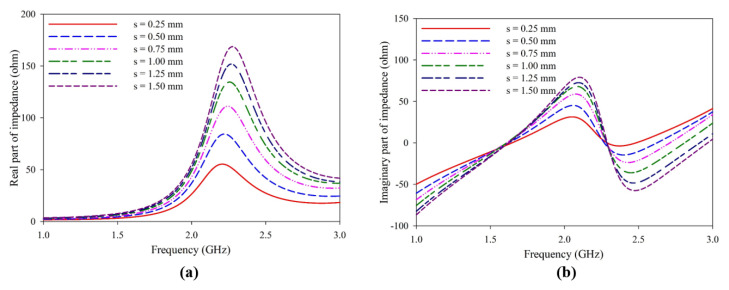
Variation of impedance of the antenna with the coupling distance ***s***. (**a**) Real part (**b**) Imaginary part.

The effect of the dimension’s length of the dipole (***L***)***,*** width of the dipole (***W***), width of the dipole arm (***d***), and coupling distance (***s***) is studied in detail to determine the antenna optimization. The dimension ***L*** = ***W*** is taken throughout the study. Coupling parameter ***s*** is optimized for the best impedance matching. As the coupling distance ***s*** increases, the impedance changes, increasing the mismatch at the desired frequency, as presented in Figure 3a. The imaginary part of impedance also varies with the coupling distance; at resonance, the circuit becomes more and more inductive, as shown in Figure 3b. 

**Figure 4:**
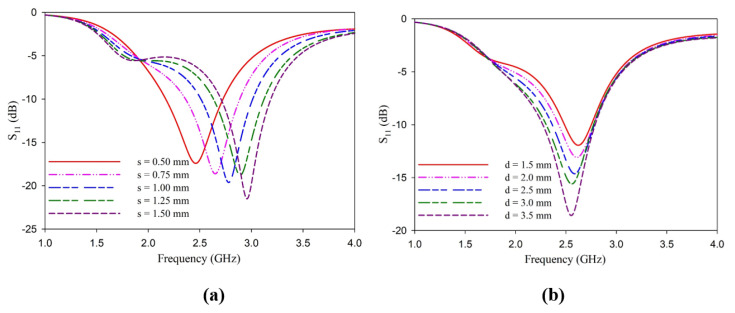
Variation of reflection coefficient of the antenna (**a**) with the coupling distance ***s*** (**b**) with the strip width ***d***.

**Figure 5:**
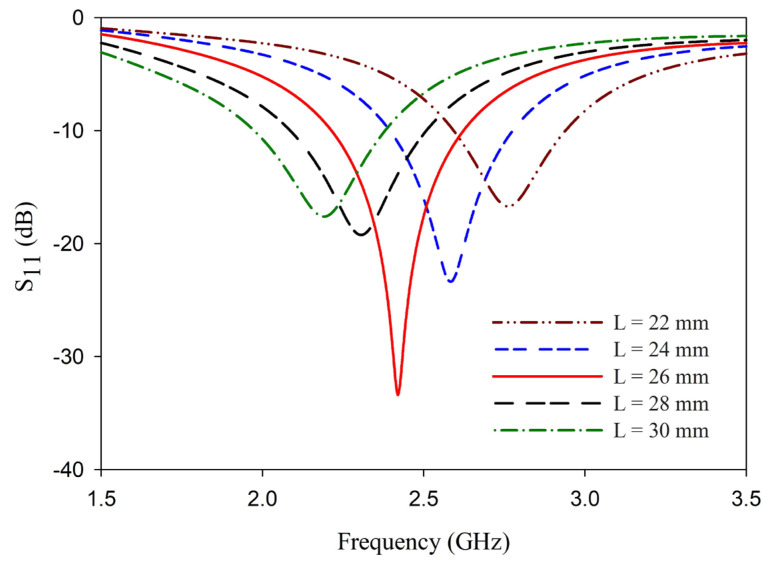
Variation of reflection coefficient of the antenna with the length of the dipole ***L.***

Antenna resonance also shifts as the coupling distance is varied, as portrayed in Figure 4a. The strip width d is also optimized for the best performance of the antenna, and the results are illustrated in Figure 4b. The coupling distance has more influence on the antenna impedance and resonance frequency, while the strip width influences the depth of the reflection coefficient, thus generating the mismatch. Considering all these factors, together with the ease of fabrication, the coupling distance is fixed at 0.5 mm, and the strip width is fixed at 3 mm. An analysis to find the dependence of the length of the dipole with ***L*** = ***W*** in reflection characteristics is also carried out. This analysis is presented in Figure 5.

**Figure 6:**
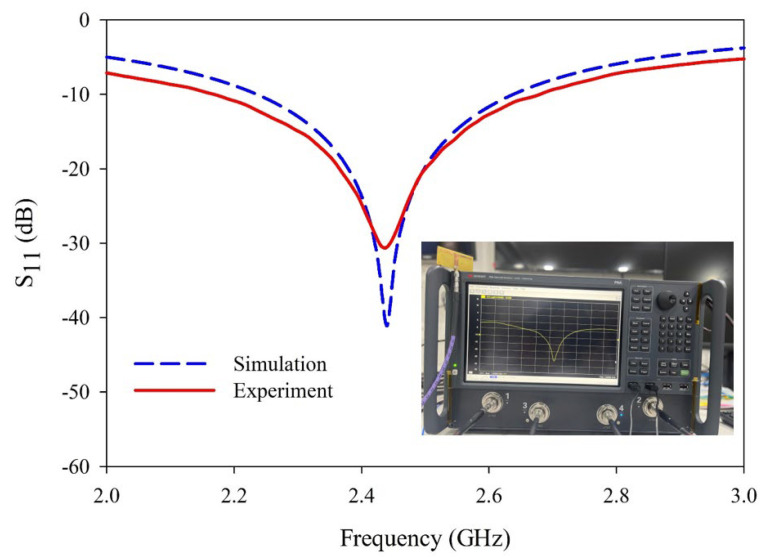
Variation of reflection coefficient of the antenna with frequency.

In this analysis, the physical dimension of the antenna is optimized as ***L*** = ***W*** = 26mm, ***d*** = 3 mm, and ***s*** =0.5 mm. The reflection characteristics of this L-shaped planar dipole antenna are depicted in Figure 6.

**Figure 7:**
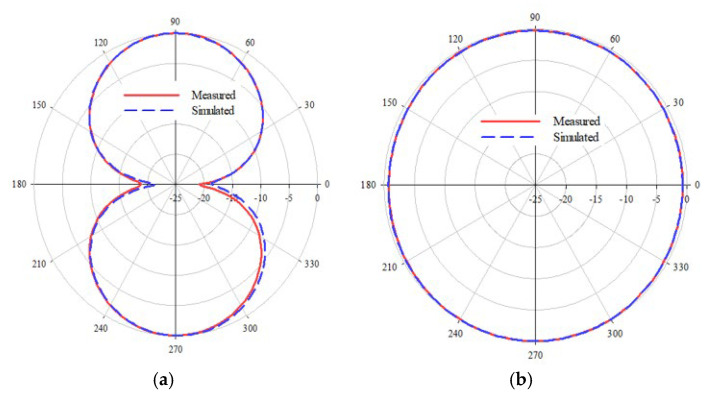
Radiation pattern in the two principal planes. (**a**) Elevation. (**b**) Azimuth.

**Figure 8:**
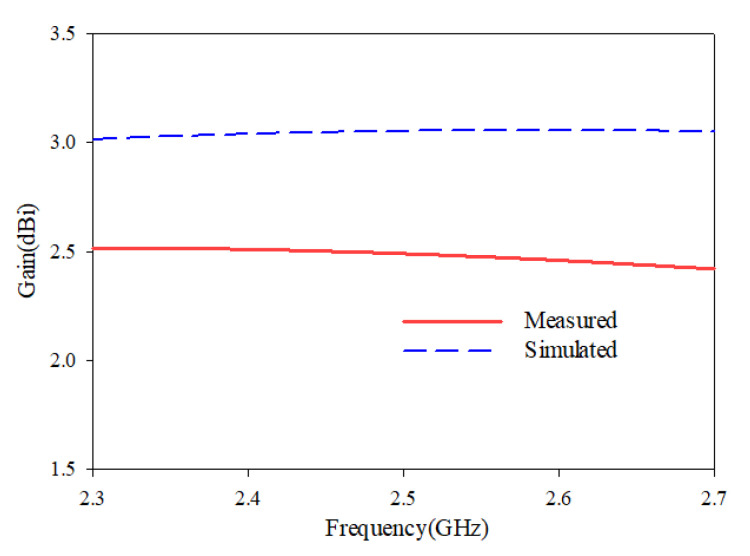
Gain of the antenna.

The radiation characteristics of the L-shaped coplanar strip dipole are also studied at the resonance frequency, and the radiation pattern of the antenna is depicted in Figure 7. The simulated and measured gain is shown in Figure 8. In the analysis and the results plotted in Figure 3, Figure 4, Figure 5, Figure 6, Figure 7 and Figure 8, the proposed antenna can be concluded as an efficient radiator and can be used for communication purposes where omnidirectional characteristics are needed. The next section discusses the application of this antenna as a sensor for detecting adulterants in liquid food samples.

## 4. L-Shaped Coplanar Strip Dipole Antenna as Sensor

**Figure 9:**
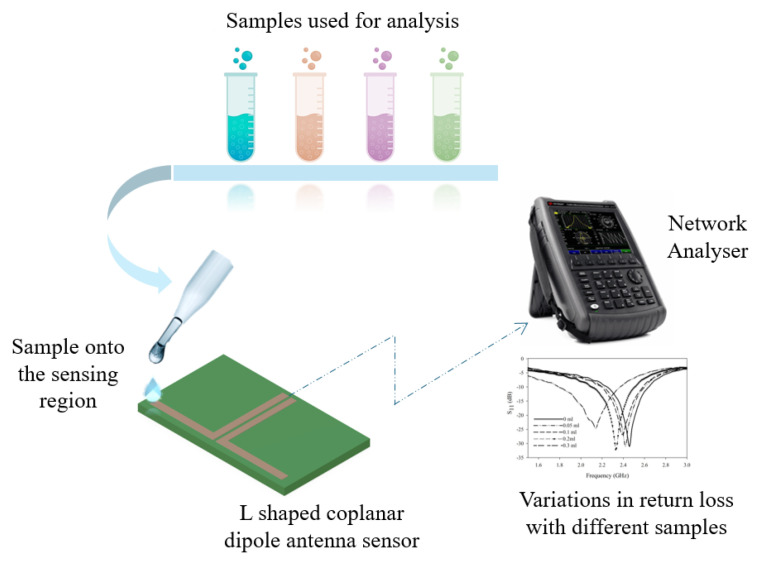
Methodology adopted for sensing.

The geometry of the L-shaped dipole antenna makes it work as an effective sensor in many scenarios. This work explores the use of the antenna as a sensor for adulteration detection. The methodology adopted for the sensing mechanism is presented in Figure 9.

**Figure 10:**
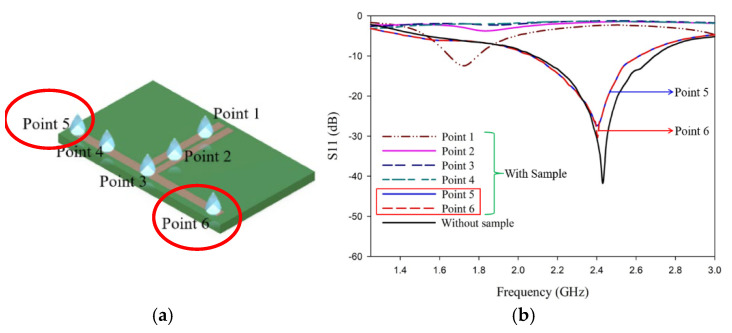
Sensing points tested on the antenna. (**a**) Positioning of liquid samples. (**b**) Variation of reflection coefficient with position of the sample.

The position of the droplets on the sensor influences the resonance frequency [18]. The discontinuity at the end of the arm is found to be more sensitive to the sample placement, which is clear in the electric field distribution shown in Figure 10a, and is validated by the experiment. The liquid sample (0.2 mL) is placed in six different regions, as marked in Figure 10b for the antenna sensor, to find the optimum position to give variation in frequency with impedance matching. 

**Figure 11:**
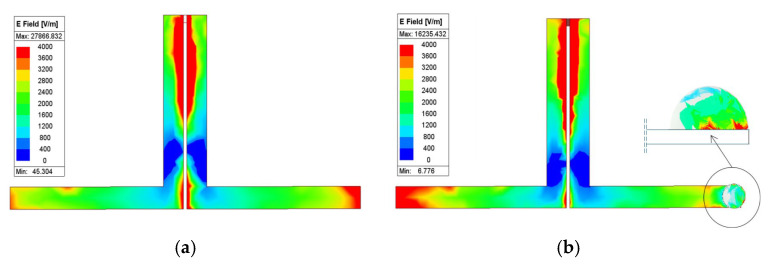
Electric field distribution. (**a**) Without adulterant. (**b**) With adulterant.

The marked points 5 and 6 have a greater field concentration, which couples more energy to the liquid to be tested. Three different samples are tested in this way to confirm the sensing region using an experiment. The presence of a liquid droplet field variations on the dipole arm. Figure 11 plots the electric field intensity in the antenna sensor. When we place a droplet in the sensing zone, the field becomes electromagnetically coupled to the droplet, as shown in Figure 11b, altering the near field and thereby changing the reflection characteristics.

On the sensing region of the antenna, different quantities (0.1 to 0.5 mL) of unadulterated milk are placed, and the behavior in the frequency spectrum is studied. As the volume increases, the spread of the sample increases, which varies the dielectric constant and creates more perturbation in the field. 

**Figure 12:**
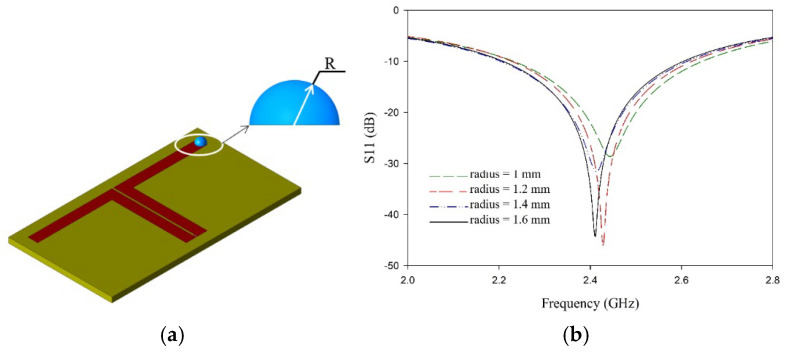
Resonance shift in antenna sensor with volume of the sample. (**a**) Droplet positioning in the simulation model. (**b**) Variation of resonance with droplet radius.

The resonance shift is minimum when the droplet radius is less than or equal to 1 mm, which is 33% lesser than the 3 mm wide L-dipole arm. Beyond this limit, the shift is linear for radii up to 1.2 times the dipole’s width, after which the dispersion is lost, and the shift remains constant. The variation in frequency with the volume of the droplet (radius) is presented in Figure 12. With this optimized position and droplet size, further experiments are carried out.

## 5. Results and Discussion

**Figure 13:**
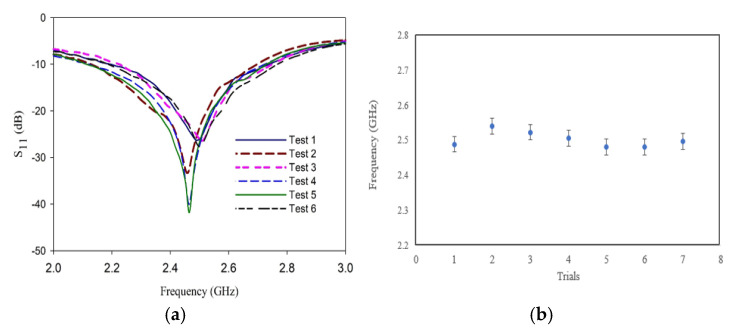
Repeatability analysis of the antenna. (**a**) Resonance frequency. (**b**) Standard error.

Section 3 presents the simulated and experimental results obtained when the L-shaped coplanar strip is used as an antenna. The experimental and simulated results are in good agreement. The antenna offers an omnidirectional radiation pattern. The reflection characteristics of the antenna are repeated to ensure that when used as a sensor, the benchmarking does not fail. The repeated output is presented in Figure 13.

**Figure 14:**
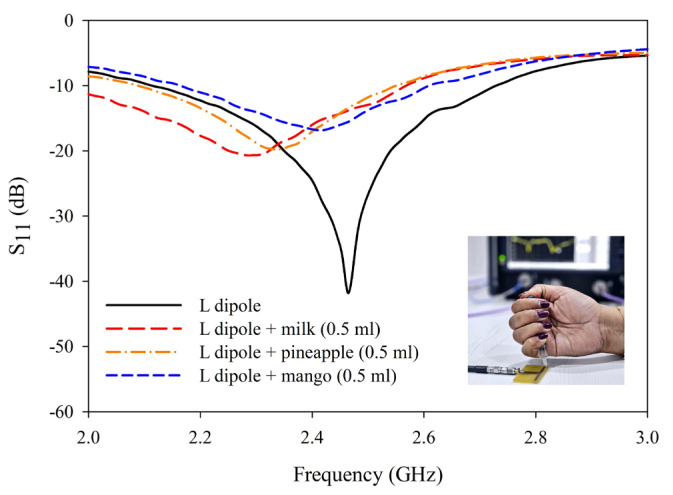
Resonance shift in antenna sensor with different unadulterated samples.

To study the performance of the antenna sensor, unadulterated samples of milk, pineapple juice, and mango juice are placed in the sensing region. In each case, the shift in resonance is noted for a different volume of the sample. As depicted in Figure 14, a significant shift in resonance is observed with different samples. Once the working of the sensor is established, three different liquid samples with and without adulterants are studied in detail. In the present study, hydrogen peroxide is taken as an adulterant [6]. 

**Figure 15:**
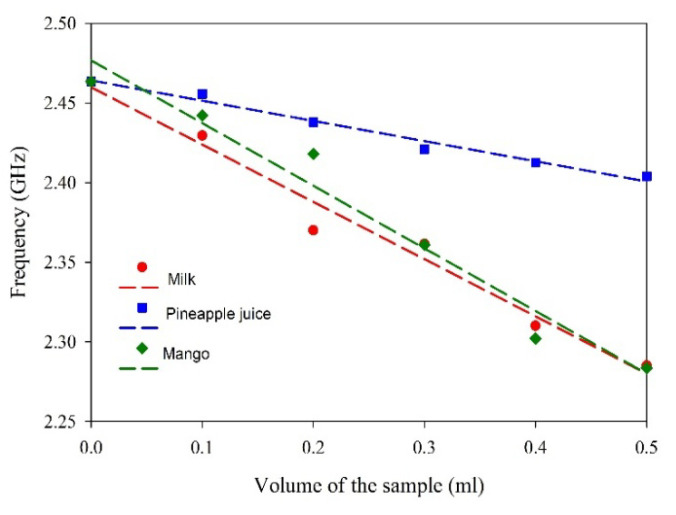
Linearity in the sensor measurements.

The influence of different volumes of milk, pineapple juice, and mango juice is analyzed in terms of the reflection characteristics of the sensor and is presented in Figure 15. The trend line in each of the cases is studied and presented as broken lines of the same color together with the experimental data. The trend line provides the linearity and sensitivity of the proposed sensor and is presented in Table 1. The experiment’s results show a sensitivity of −359.9 MHz/mL for milk and −393.5 MHz/mL and −126.9 MHz/mL for pineapple juice without the addition of adulterant. The sensor responses are linear for all the samples studied.

To study the influence of adulterants, the amount of sample tested is kept constant with the addition of the adulterant. Thus, x ml of sample will have 50% pure liquid sample and 50% adulterant. The sensor responses are shown in Table 2.

**Figure 16:**
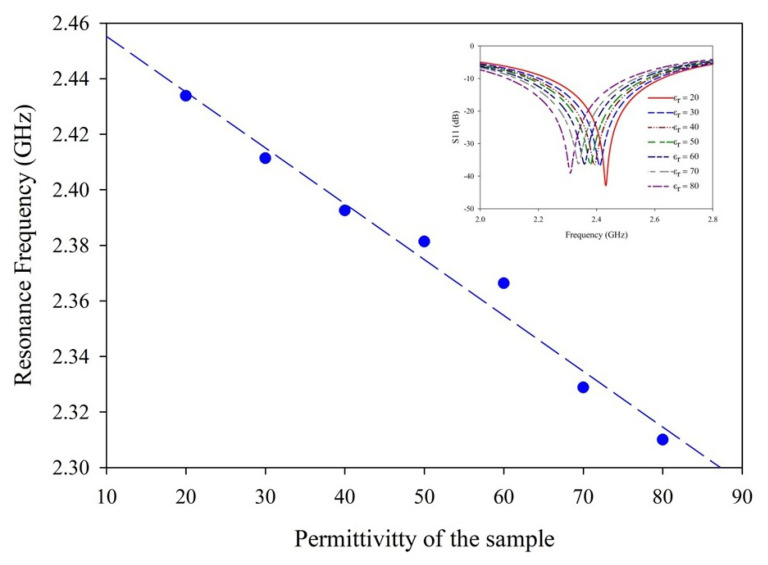
Variation of resonance with the permittivity of the sample.

The variance in frequencies is comparable, resulting in distinguished sensitivities. Furthermore, the use of adulterants increased the sensitivity. The regression analysis in Table 1 and Table 2 indicates the sensitivity of the sensor for different samples. For unadulterated milk and mango juice, the sensitivity is −359.9 MHz/mL and −393.5 MHz/mL, respectively. However, the adulterated samples show a significant sensitivity of −491 MHz/mL and −766.3MHz/mL, respectively. The variation in the sensitivity range can be explained by variation in dielectric properties (Table 3). The addition of hydrogen peroxide into the sample causes variations in the dielectric constant, which affects the resonant frequency of the sensor, as depicted in Figure 16 (R^2^ = 0.978). 

The dielectric constants of milk, pineapple juice, and mango juice are 57, 73.7, and 32.5, respectively [18,33,34]. Table 3 shows how the addition of hydrogen peroxide (ε_r_ = 84.2) changes the dielectric constant of the mixture [33] and is calculated as per Equations (1) and (2).

(1) $$\varepsilon_{mix}=V_{fs}\varepsilon_{s}+V_{fa}\varepsilon_{a}$$

(2) $$\Delta \varepsilon =\frac{\varepsilon_{mix}-\varepsilon_{s}}{\varepsilon_{s}}$$

where

$\varepsilon_{mix}$ is the dielectric constant of the mixture (sample added to adulterant).
$V_{fs}$ is the volume fraction of the unadulterated sample.
$V_{fa}$ is the volume fraction of the adulterant.
$\varepsilon_{s}$ is the dielectric constant of the unadulterated sample.
$\varepsilon_{a}$ is the dielectric constant of the adulterant.

Table 3 shows the percentage change in the dielectric constant resulting from the addition of 10% adulterant. When hydrogen peroxide is added to mango juice, a 15.9% shift in its dielectric constant is observed resulting in a high sensitivity of −766.3 MHz/mL (Table 2).

**Figure 17:**
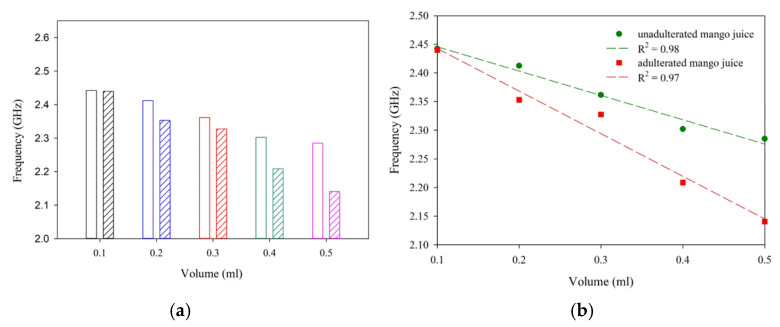
Sensor response with different volumes of droplets (mango juice and H_2_O_2_). (**a**) Data comparison. (**b**) Regression analysis.

**Figure 18:**
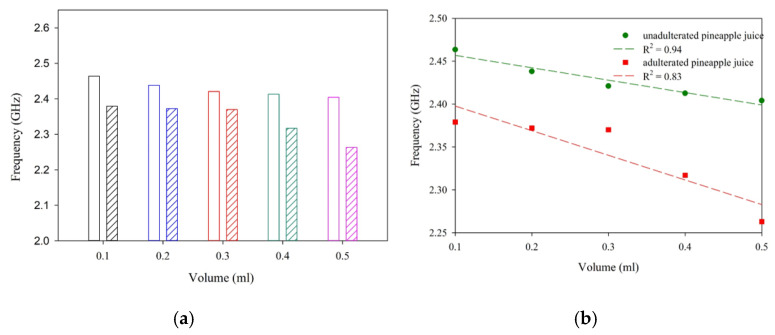
Sensor response with different volumes of droplets (pineapple juice and H_2_O_2_). (**a**) Data comparison. (**b**) Regression analysis.

**Figure 19:**
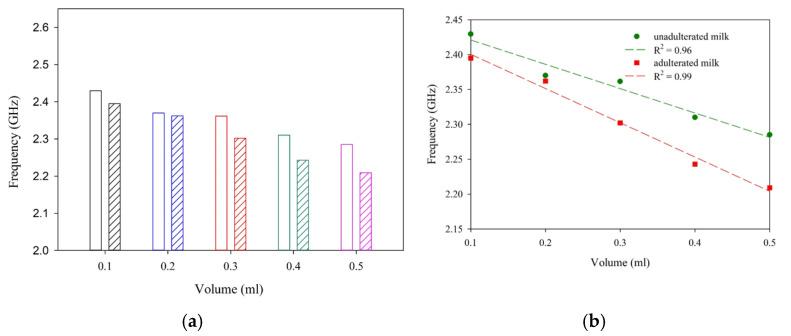
Sensor response with different volumes of droplets (milk and H_2_O_2_). (**a**) Data comparison. (**b**) Regression analysis.

Figure 17, Figure 18 and Figure 19 illustrates the shifts in resonance frequency with the volume of the sample. Here, an equal amount of juice and adulterant is used for the experimental analysis. In this study, the net amount of the sample quantity with and without adulterants varied from 0.1 to 0.5 mL. A 1:1 mixing ratio is maintained without alternating the net quantity of the sample. With 50% pure liquid and 50% adulterant, the frequency shift is significant in the samples studied. This helps us in the detection of H_2_O_2_ in the given sample. 

To identify the percentage of adulterants in each sample, another set of experiments was conducted. These experiments are performed by adding 2% to 10% H_2_O_2_ to 3 ml of pineapple juice. The variation of the dielectric constant is calculated (Table 4) for these mixing ratios. 

**Figure 20:**
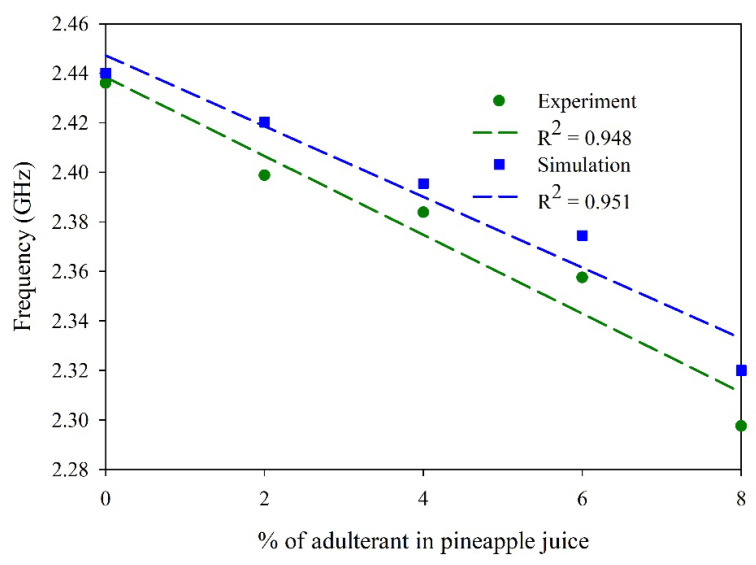
Sensor response with adulterant (pineapple juice and H_2_O_2_).

The experiments are carried out with these mixing ratios and compared with the simulated results. The obtained frequency response is as depicted in Figure 20. The experiments show a sensitivity of −159 MHz/% adulterant concentration. The simulations accounts for the variation in the dielectric constant alone. However, considering the loss tangent and conductivity variations, we can further optimize the FEA model for better prediction. Thus, the proposed sensor is proved to be an effective tool to identify the presence of H_2_O_2_ in the given food sample. A comparison of the proposed sensor with the referred work in the area of liquid sensing is carried out and presented in Table 5.

**Table 5:** Comparison of the sensor with existing works reported in literature.

Sl. No:	Type of Antenna Sensor	Substrate	Size (mm^3^)	Frequency of Operation (GHz)	Sensitivity
1	Present work	ε_r_ = 4.4 h = 1.6 mm	52.5 × 28 × 1.6	2.46	159 MHz/% pineapple juice with H_2_O_2_
2	[18]	ε_r_ = 4.4 h = 1.6 mm	38.8 × 38.8 × 1.6	2.48	0.27
3	[25]	ε_r_ = 4.4 h = 2.5 mm	10 × 10 × 1.56	13.3	28%
4	[26]	ε_r_ = 4.5 h = 2.5 mm	80 × 80 × 31.5	6.9	0.038
5	[27]	ε_r_ = 4.4 h = 1.5 mm	35 × 25 ×1.6	2.4	0.214
6	[28]	ε_r_ = 4.4 h = 1.5 mm	22.84 × 10.18 × 11.57	8 −12.5	0.85
7	[29]	ε_r_ = 3.5 h = 0.76 mm	80 × 80 × 0.76	2.653	0.45
8	[30]	ε_r_ = 4.4 h = 1.6 mm ε_dr_ = 9.8 h = 6.35 mm	80 × 80 × 7.95	2.25	8.75 MHz/ε_r_
9	[31]	ε_r_ = 2.2 h = 3.175 mm	90 × 90 × 3.175	4.4	-
10	[32]	ε_r_ = 2.2 h = 0.8 mm	40 × 15 × 0.8	2.6	0.27

## 6. Conclusions

A significant concern confronting the food industry today is the utilization of chemical adulterants as preservatives to prolong shelf life. Evaluating the quality of food products and verifying the presence of adulterants within permissible limits would be laborious in our everyday routine. Hydrogen peroxide is a common adulterant employed in certain countries to prolong the shelf life of dairy products, and its usage is prohibited in others. To this end, the findings of this study may offer a feasible and efficient approach for detecting hydrogen peroxide in liquid food samples. This work presents a comprehensive technique for the design and testing of an RF sensor (Co-planar L-Dipole antenna) to detect hydrogen peroxide in liquid food products, including milk, mango juice, and pineapple juice. The sensor has been designed for the 2.45 GHz frequency band, which is significant in antenna communication. The antenna sensor’s sensing capabilities were numerically evaluated and experimentally tested. The presence of adulterants in the samples induces a shift in the fundamental resonant frequency of the antenna, which constitutes the operational principle of the antenna sensor. The maximum sensitivity of -766.3 MHz/mL is achieved with pineapple juice containing 10% hydrogen peroxide. The authors are working on the possibility of making this sensor a compact gadget module with wireless sensing capabilities, which will aid in detection in the future.

## Figures and Tables

**Table 1:** Regression analysis of different samples without adulterant.

Sample	R^2^	Sensitivity (MHz/mL)
Milk	0.96	−359.9
Mango juice	0.98	−393.5
Pineapple juice	0.94	−126.9

**Table 2:** Regression analysis of different samples with adulterant.

Sample	R^2^	Sensitivity (MHz/mL)
Milk with H_2_O_2_ (50% each)	0.99	−491
Mango juice with H_2_O_2_ (50% each)	0.97	−766.3
Pineapple juice with H_2_O_2_ (50% each)	0.83	−287.1

**Table 3:** Sensitivity related to dielectric constant of the mixture.

Item	Concentration (%)		Δε
	0%	10% H_2_O_2_	$\Delta \varepsilon =\frac{\varepsilon_{mix}-\varepsilon_{s}}{\varepsilon_{s}}\times \mathrm{100\%}$
Milk	57	59.72	4.77%
Pineapple juice	73.7	74.75	1.42%
Mango juice	32.5	37.67	15.9%

**Table 4:** Dielectric constant and frequency variation of all samples tested with H_2_O_2_.

Sl No			100% Sample	98% Sample	96% Sample	94% Sample	92% Sample	90% Sample
1	Total volume (*V*)	(ml)	3					
2	Volume of sample (*V*_s_)	(ml)	3	2.94	2.88	2.82	2.76	2.7
3	Volume of adulterant (*V*_a_)	(ml)	0	0.06	0.12	0.18	0.24	0.3
4	Volume fraction of sample (*V*_fs_)		1	0.98	0.96	0.94	0.92	0.9
5	Volume fraction of adulterant (*V*_fa_)		0	0.02	0.04	0.06	0.08	0.1
6	ε_mix_ (Pineapple)		73.7	73.91	74.12	74.33	74.54	74.75
7	ε_mix_ (Milk)		57	57.544	58.088	58.632	59.176	59.72
8	ε_mix_ (Mango juice)		32.5	33.534	34.568	35.602	36.636	37.67

## Data Availability

Data are contained within the article.
